# On optimal control policy for probabilistic Boolean network: a state reduction approach

**DOI:** 10.1186/1752-0509-6-S1-S8

**Published:** 2012-07-16

**Authors:** Xi Chen, Hao Jiang, Yushan Qiu, Wai-Ki Ching

**Affiliations:** 1 Advanced Modeling and Applied Computing Laboratory, Department of Mathematics, The University of Hong Kong, Hong Kong

## Abstract

**Background:**

Probabilistic Boolean Network (PBN) is a popular model for studying genetic regulatory networks. An important and practical problem is to find the optimal control policy for a PBN so as to avoid the network from entering into undesirable states. A number of research works have been done by using dynamic programming-based (DP) method. However, due to the high computational complexity of PBNs, DP method is computationally inefficient for a large size network. Therefore it is natural to seek for approximation methods.

**Results:**

Inspired by the state reduction strategies, we consider using dynamic programming in conjunction with state reduction approach to reduce the computational cost of the DP method. Numerical examples are given to demonstrate both the effectiveness and the efficiency of our proposed method.

**Conclusions:**

Finding the optimal control policy for PBNs is meaningful. The proposed problem has been shown to be $\sum_{2}^{p}\text{ - hard}$. By taking state reduction approach into consideration, the proposed method can speed up the computational time in applying dynamic programming-based algorithm. In particular, the proposed method is effective for larger size networks.

## Background

An important goal for studying genetic regulatory network is to understand the gene behavior and to develop optimal control policy for potential applications to medical therapy. While many models have been proposed for modeling gene regulatory networks, Boolean Networks (BNs) [1-3] and thier extension Probabilistic Boolean Networks (PBNs) [4] have received much attention. Because they form a class of models which can capture the logical interactions of genes and they are also effective in modeling pathways for drug discovery [5]. Recently applications in medical treatment for Parkinson's disease can also be found in [6]. In fact, a PBN can be considered as a collection of BNs driven by a Markov chain and therefore its dynamics and behavior can be studied by using Markov chain theory. For reviews on BNs and PBNs, we refer interested readers to [7-9] and the references therein.

Many methods in control theory are available for the intervention of PBNs. A gene control model has been proposed in [10]. The control model is formulated as a mixed integer programming problem and it aims at driving the PBN from the undesirable states to the desirable ones. A class of PBN control problems with hard constraints has been proposed in [11,12]. The motivation of the control model is to reduce the side-effects of medical treatment. In [11], hard constraints are included in the optimal control problem and an approximation method is then proposed in [12] to obtain the optimal controls efficiently.

Datta et al. [13] proposed an external intervention method based on optimal control theory. In their work, genes are classified as internal nodes and external nodes (control nodes). One can intervene the values of internal nodes in some desirable manner by controlling the values of certain external nodes. By defining the control cost for each control input and terminal cost for each state, the problem is to find a sequence of control inputs that leads the network into desirable states at the terminal step with minimum average cost. The classical technique of dynamic programming is then employed to solve the optimal control problem.

Chen et al. [14] then consider an external intervention problem based on optimal control theory and dynamic programming. Given the terminal cost of each state, the objective is to drive the network into the state with the maximum cost being minimized by applying external controls. The problem is important in the view of medical therapy because patients/organisms would like to minimize the damage even for the worst case. They proved that both minimizing the maximum cost and minimizing the average cost are $\sum_{2}^{p}\text{ - hard}$. A dynamic programming-based algorithm is then proposed for finding a control sequence that minimizes the maximum cost in control of PBN. The above dynamic programming-based methods still have high computational complexity. The size of the underlying transition probability matrix increases exponentially with the number of nodes in the PBN. To tackle this problem a possible remedy is to consider network reduction approach.

Several reduction methods have been proposed recently. In [15], a CoD-based reduction algorithm is introduced. Coefficient of Determination (CoD) helps to evaluate the influence of a candidate node for deletion on the target node and find the optimal candidate node for deletion. The proposed algorithm can well preserve the attractor structure and long-run dynamics of the original network.

Qian et al. [16] proposed a state reduction method by considering deleting states directly. Instead of deleting the nodes in a network, they delete the out-most states having less influence to the network. Here we consider a transition probability-based reduction strategy. This strategy is easy to implement as we do not need to compute the stationary distribution of the PBN beforehand.

We consider the problem of minimizing the maximum cost in control of PBN and we employ transition probability-based reduction strategy to reduce the network complexity of a PBN. We show that under some condition and in many of our numerical examples, the optimal control sequence obtained from the reduced network is the same as the one in the original network. Then we apply the dynamic programming-based algorithm to the reduced network. The computational complexity of dynamic programming-based algorithm when applied to the original network is *O*(2^*n*^) (depending on the number of network states) when the number of control nodes *m *and the number of steps *M *are fixed. When our state reduction method is applied, the computational complexity is reduced to *O*(*|R|*), where *R *is the set of states after reduction.

The remainder of the paper is structured ae follows. We first give a brief review on PBNs and the dynamic programming method. We then introduce our state reduction approach together with some theoretical results to support our proposed approach. Numerical examples are given to demonstrate both the effectiveness and the efficiency of our proposed method. Finally some discussion will be given to conclude the paper.

### A brief review on BNs and PBNs

A BN consists of a set of *n *nodes (genes) as follows: {*v*_1_,*v*_2_,..., *v_n_*}, *v_i_*∈ {0,1} and a set of Boolean functions denoted by {*f*_1_, *f*_2_,..., *f_n_*}. Each *v_i_*(*t*) is defined as the state of node *i *at time *t*. The rules of regulatory interactions among nodes are then represented by the Boolean functions: *v_i_*(*t + *1) *= f_i_*(*v_i_*_1_, *v_i_*_2_,..., *v_ik_*) where {*v_i_*_1_,*v_i_*_2_,...,*v_ik_*} are input nodes of *f_i_*, and they are called parent nodes of node *v_i_*. We define *IN*(*vi*) = {*v_i_*_1_, *v_i_*_2_,..., *v_ik_*}. The number of parent nodes to *v_i_*is called the *in-degree *of *v_i_*. The largest in-degree of {*v*_1_, *v*_2_,..., *v_n_*} is called the *maximum in-degree *of BN and is denoted by *K*.

Since BN is a deterministic model, a stochastic model is more preferable due to the measurement noise in inferring a gene regulatory network. A stochastic version of BN, PBN [4,9] is then introduced to cope with the weakness. A PBN can be regarded as an extension of BN to a probabilistic setting. In a PBN, each node *v_i_*has a set of Boolean functions:

(1) $$\left\{f_{1}^{\left(i\right)},f_{2}^{\left(i\right)},\ldots ,f_{l\left(i\right)}^{\left(i\right)}\right\}.$$

The state of *v_i_*at time *t + *1 is predicted by one of the Boolean functions in (1) with selection probabilities $c_{j}^{\left(i\right)}$. Here

$$\overset{l\left(i\right)}{\underset{j=1}{\sum }}c_{j}^{\left(i\right)}=1,\;\;c_{j}^{\left(i\right)}\geq 0\;\;\text{for}\;j\;=1,2,\ldots ,l\left(i\right).$$

A PBN can be regarded as a finite collection of BNs over a fixed set of nodes, where each BN has a fixed set of Boolean functions $f_{j}=\left\{f_{j_{1}}^{\left(1\right)},f_{j_{2}}^{\left(2\right)},\ldots ,f_{j_{n}}^{\left(n\right)}\right\}$. The BN having Boolean function set **f_j_**(*j = *1,2,...,*N*) is called the *j*th BN. At each time step *t*, the selection process of Boolean functions is assumed to be independent, and the selection probability is given by $q_{j}=c_{j_{1}}^{\left(1\right)}c_{j_{2}}^{\left(2\right)}\ldots c_{j_{n}}^{\left(n\right)},j=1,2,\ldots ,N$ and the states of {*v*_1_(*t + *1),*v*_2_(*t *+ 1),...,*v_n_*(*t + *1)} is predicted by the Boolean function set **f_j_**. Then we introduce the decimal representation of states. Suppose the current state is {*v*_1_(*t*), *v*_2_(*t*),..., *v_n_*(*t*)}, we define

$$w\left(t\right)=1+\overset{n}{\underset{i=1}{\sum }}2^{n-i}v_{i}\left(t\right).$$

Since *v_i_*(*t*) ∈ {0,1}, *w*(*t*) can take any integral value in [1, 2^*n*^].

The dynamics of a PBN can be studied by using Markov chain theory, see for instance [17]. The one-step transition probability can be represented by using the transition probability matrix *A *where each entry *A_ij _*is given by

(2) $$A_{ij}=\underset{k\in I}{\sum }q_{k},\;i,j=1,2,\ldots ,2^{n}.$$

Here *i = w*(*t + *1) and *j = w*(*t*) and  is set of BNs that the network can enter state *i *from state *j*. We remark that *A *is a column stochastic matrix, i.e., $\sum_{j=1}^{2^{n}}{\text{A}}_{ij}=1$.

## A review on dynamic programming

In this section, we first introduce several definitions to facilitate the discussion. We then introduce the dynamic programming-based algorithm. Suppose a PBN has a set of internal nodes {*v*_1_,*v*_2_,...,*v_n_*} which is the same as the node set defined in the previous Section, and a set of external nodes (control nodes) {*v_n+_*_1_,*v_n+_*_2_,...,*v_n+m_*}. At time *t+ *1, the states of *v_i_*, *i = *1,2,..., *n *are predicted by $\upsilon_{i}\left(t+1\right)=f_{j}^{\left(i\right)}\left(\upsilon_{i1},\upsilon_{i2},\ldots ,\upsilon_{ik}\right)$ where *v_ik_* can be either an internal node or an external node. This provides a possible way for intervening the states of internal nodes by controlling the values of external nodes. To facilitate our discussion, we adopt the following state representation of the network and define

(3) $$z_{t}=1+\overset{n}{\underset{i=1}{\sum }}2^{n-i}\upsilon_{i}\left(t\right)$$

to be the state of network. Then we define control input as

(4) $$\upsilon_{t}=1+\overset{m}{\underset{i=1}{\sum }}2^{m-i}\upsilon_{n+i}\left(t\right).$$

Here we are interested in the following problem: Minimizing the maximum cost in control of PBN.

Given the terminal cost *C*(*z_M_*) for each state *z_M_*∈ {1,2,...,2^*n*^} at terminal time step *M*, find a sequence of control input *u*_0_,*u*_1_,...,*u_M_*such that starting from the given initial state the network will enter into the state with minimized maximum cost at time step *M*. In [14], a dynamic programming-based method is proposed for the above problem:

**Step 0: **Set *t = M*;

*J*(*z_M_*, *h_M_*) *= C*(*z_M_*) for all *h_M _= *{0,..., *M*}.

**Step 1**: *t : = t *-1.

**Step 2**: For any *z_t_*∈ {1,..., 2^*n*^} and *h_t_*∈ {0,..., *M*}, compute

$$J\text{(}z_{t}\text{,}h_{t}\text{) = }\underset{\upsilon_{t}\in \left\{1\text{,}\ldots \text{,}2^{m}\right\}}{\text{min}}\left\{\begin{array}{c} \underset{z_{t+1}\in F\left(z_{t},u_{t}\right)}{\text{min}}J\left(z_{t+1},h_{t}\right),\;\text{if}\;u_{\text{t}}=u\left(z_{t+1},h_{t}\right),\\ \underset{z_{t+1}\in F\left(z_{t},u_{t}\right)}{\text{min}}J\left(z_{t+1},h_{t}-1\right),\;\;\text{otherwise}\text{.} \end{array}\right)$$

and

$$u\left(z_{t},h_{t}\right)=\text{arg}\underset{u_{t}\in \left\{1,\ldots ,2^{m}\right\}}{\text{min}}\left\{\begin{array}{c} \underset{z_{t+1}\in F\left(z_{t},u_{t}\right)}{\text{max}}J\left(z_{t+1},h_{t}\right),\;\;\text{if}\;u_{t}=u\left(z_{t+1},h_{t}\right),\\ \underset{z_{t+1}\in F\left(z_{t},u_{t}\right)}{\text{max}}J\left(z_{t+1},h_{t}-1\right),\;\;\text{otherwise}. \end{array}\right)$$

**Step 3**: If *t >*0, go back to **Step 1**; Otherwise, stop.

## The state reduction approach

In this section we propose our state reduction method.

### Transition probability-based state reduction strategy

Due to the high network complexity of a PBN, one has to deal with matrices of huge size which increases exponentially with the number of internal nodes. Network reduction is therefore an important issue to be addressed in this situation. In [16], a transition probability-based state reduction strategy is proposed. In a PBN, we consider all attractor states and initial state as critical states, and they are preserved during state reduction. A state *i *can be deleted if the following equation is satisfied:

(5) $$\overset{2^{n}}{\underset{j=1}{\sum }}A_{ij}<\xi$$

where *ξ >*0 is a parameter to be predetermined. The value of *ξ *depends on perturbation probability and it is usually not large. When we consider PBNs without perturbation, Equation (5) can be rewritten as

(6) $$\overset{2^{n}}{\underset{j=1}{\sum }}A_{ij}=0.$$

Which means that the network will never enter state *i *from other states. Hence, deleting state *i *will not influence the steady-state distribution of the network.

### The dynamic programming-based algorithm on the reduced network

Since the computational complexity of the dynamic programming is *O*(2^*n*^) when the number of control nodes *m *and the number of steps *M *are fixed, using state reduction may reduce the computation complexity to *O*(*|R|*), where *R *is the set of states after reduction. It is straightforward to see that we have the following proposition.

**Proposition 1 ***The result of dynamic programming-based algorithm on the reduced network will be the same as the one on the original network*.

It is straightforward to see that, starting from the initial state, the network will never enter into transient states to be deleted. Therefore the network will never stop at those states at the terminal time step. This means that the deleted states will not be included in the optimal route, and the cost of deleted states will not be counted. Hence deleting these transient states will not influence the result obtained from the DP method when applied to the reduced network.

Based on transition probability-based strategy, one can iteratively delete those transient states until all the remaining states are critical states. In each step, we need to update the transition matrix for the reduced network by deleting the corresponding row and column from the transition matrix. After making the reduction, one can get a reduced network with a set of states *R *and a *|R|*-by-*|R| *transition probability matrix *B*. Then we can apply dynamic programming-based algorithm on the reduced network. In the following, we give a theoretical result on the reduction method when the indegree of the network is one.

### An analysis of the reduction method when indegree *K *= 1

In a PBN of *n *genes, there are totally 2*n *states in the network. When *K = *1, it means that each gene is controlled by only a single gene. Table 1 gives an example when the number of genes is two. It is straightforward to compute the number of all the possible BNs which is actually 4*×*4 = 16.

**Table 1:** All the possible BNs for 2 genes when *K *= 1

States	f_1_				f_2_			
	***f*_11_**	***f*_12_**	***f*_13_**	***f*_14_**	***f*_21_**	***f*_22_**	***f*_23_**	***f*_24_**

00	0	1	0	1	0	1	0	1
01	0	1	1	0	0	1	1	0
10	1	0	0	1	1	0	0	1
11	1	0	1	0	1	0	1	0

In general, we can also compute the number of all the possible BNs for *n *genes: (2*n*)^*n*^. For example, from Table 1, one can calculate there are totally (2 *× *2)^2 ^= 16 networks with 2^2 ^*× *2^2 ^sizes. When every row contains 1, it means the number of nonzero rows is 2^2^. To satisfy this condition, we have to choose 2 genes as parent genes and consider every gene has two possible states. Thus, we can deduce that the number of such networks is $A_{\text{2}}^{\text{2}}\times 2\times 2=8$ where $A_{r}^{n}=n\text{!/}r\text{!}$. But when the number of nonzero rows is 2^1^, we just select only one gene as the parent gene and the corresponding selected possibilities are $C_{1}^{2}$ where $C_{r}^{n}=n\text{!}/\left(r\text{!}\left(n-r\right)\text{!}\right)$. Since for each gene, there exist two states to be selected. Therefore, the total number of such networks is $C_{1}^{2}\times 2\times 2=8$. In determination of the linear combination of BNs for construction of PBN, the intrinsic structure of BNs plays an utmost role. Here we study the distribution of nonzero rows in BNs and we give the following distribution theory.

**Proposition 2 **When the indegree of a BN is one, the distribution of zero row is given in Table 2. Moreover, the probability of getting a BN having no zero row decreases to zero at a fast rate of $\frac{n\text{!}}{n^{n}}$ as the number of genes n increases to infinite.

**Table 2:** Distribution of number of nonzero rows in BNs when *K *= 1

Number of nonzero rows in BN	Number of BNs in all the (2*n*)^*n*^BNs
2^*n*^	*n*!2^*n*^

2^(*n-k*)^*k *= 1,2,...,(*n *- 1)	$n!C_{n}^{n-k}2^{n}\sum_{j=1}^{k}C_{n-k}^{j}\sum_{\underset{A_{i>2,\;i=1,2,\cdots ,j}}{\sum_{i=1}^{j}A_{i}=k+i}}\frac{1}{A_{1}!\cdots A_{i}!}$

In Table 2, when the number of zero rows is 0, it means that there is no zero row, there are *n*!2^*n *^such kind of BNs. This means that after transition, all the states will still be visited. In calculating the number of BNs satisfying this particular condition, we should ensure that the *n *genes have *n *parent nodes. Therefore it is easy to deduce that we can have *n*!2^*n *^BNs having no zero row. As a matter of fact, if we define a function *F_dis_*for mapping the number of non-zero rows in BNs to the number of the parent nodes for a *n*-gene set, we can have

$$F_{dis}\left(2^{\left(n-k\right)}\right)=\left(n-k\right),\;k=1,2,\ldots ,n-1.$$

Therefore to compute the number of BNs when the number of non-zero rows is 2^*n-k*^, one should select *n-k *out of *n *genes as parent nodes. And that is the reason why we have $C_{n}^{n-k}$. Since the *n-k *parent nodes will fill in *n *positions, we should take all possible selection pattern into account. Then we have the double summation part for calculating the number of BNs when the number of nonzero rows is 2^*n-k*^, *k = *1,2,..., (*n *- 1).

Furthermore, since there are 2^*n *^states for *n *genes, the number of rows in BNs is 2^*n*^. One can observe that with the increase of *n*, the ratio of the number of BNs with full number of rows to the whole number of (2*n*)^*n *^BNs is decreasing fast because

$$\underset{n\to \infty }{\text{lim}}\frac{2^{n}n!}{{\left(2n\right)}^{n}}=\underset{n\to \infty }{\text{lim}}\frac{n!}{n^{n}}=0.$$

Hence this guarantees the efficiency of state reduction.

### State reduction for PBN with random perturbation

In this section we discuss the state reduction strategy for PBNs with random perturbation. Let *p *be the perturbation probability of single gene (flipping the value of single gene from 1 to 0 or 0 to 1). Suppose the current state is **v**(t), then state at the next time step is determined by the transition matrix without perturbation *A *with probability (1-*p*)^*n*^, or by randomly perturbation with probability 1-(1-*p*)^*n*^. Therefore the transition matrix with perturbation is given by

(7) $$\tilde{A}={\left(1-p\right)}^{n}A+P.$$

where *P *is the perturbation matrix [18]:

(8) $$P=\underset{n\;\text{terms}}{\underset{︸}{Q⊗Q⊗\cdots ⊗Q}}-{\left(1-p\right)}^{n}I_{2^{n}}$$

where

(9) $$Q=\left(\begin{array}{cc} 1-p & p\\ p & 1-p \end{array}\right)$$

To carry out the state reduction strategy, we need to delete all the states which can only be entered by random perturbation. Here we set the threshold for *ξ *as the row sum of *P: ξ *= 1 - (1 - *p*)^*n*^. If for some state *i*, the following inequality

(10) $$\overset{2^{n}}{\underset{j=1}{\sum }}{Ā}_{ij}\leq \xi$$

is satisfied, then we can delete the state.

Table 3 gives the reduction rates (percentage of states deleted after network reduction) for PBNs with random perturbation. In the experiment, each PBN has 4 BNs, and the maximum in-degree is *K = *2. We consider the cases *p = *0.001, 0.002, 0.005, 0.01 and *n = *6, 8,10,12. For each case, we perform the simulation for 10 times and report the average results. From Table 3, one can see that the PBNs can delete more rows when the value of perturbation probability *p *increases.

**Table 3:** Reduction Rates for PBN with Gene Perturbations

	*n *= 6	*n *= 8	*n *= 10	*n *= 12
*p *= 0.001	12.5%	37.5%	36.0%	35.4%
*p *= 0.002	42.2%	60.5%	42.8%	48.4%
*p *= 0.005	77.5%	67.8%	63.1%	63.8%
*p *= 0.010	84.9%	73.1%	77.0%	72.4%

## Results and discussions

In this section, we give some numerical examples to compare the result of dynamic programming-based algorithm on the reduced network with the one on the original network.

### A 6-gene example

We first consider a 6-node example. We consider the cases of *m *= 1,2, *N *= 2,4,8 and *K = *2, 3. The Boolean function set of PBN are randomly generated. We let *M = *20 and *C*(*z_M_*) *= z_M_*. When *m *= 1, there are 5 internal nodes and 1 control node. The original network size is 2^5^. When *m *= 2, there are 4 internal nodes and 2 control nodes. The original network size is 2^4^. Table 4 gives the numerical results for PBNs without perturbation. Table 5 gives the numerical results for PBNs with random perturbation. The second column gives the network size before and after reduction. The third column gives minimized maximum cost obtained by using the dynamic programming-based algorithm on the original and reduced network. The last column records the CPU time of running the program for dynamic programming-based algorithm before and after reduction.

**Table 4:** A 6-Node Example for PBN Without Perturbation

	Size		Cost		CPU Time (sec.)	
	
	Original	Reduced	Original	Reduced	Original	Reduced
*m*= 1						
*N *= 2	32	24	17	17	0.1276	0.0943
*K *= 2						

*m*= 1						
*N *= 2	32	26	5	5	0.1255	0.0997
*K *= 3						

*m *= 1						
*N *= 4	32	26	21	21	0.1286	0.1062
*K *= 2						

*m *= 1						
*N *= 4	32	27	19	19	0.1291	0.1085
*K *= 3						

*m *= 1						
*N *= 8	32	30	25	25	0.1355	0.1274
*K *= 2						

*m *= 1						
*N *= 8	32	32	29	29	0.1355	0.1355
*K *= 2						

*m *= 2						
*N *= 2	16	11	9	9	0.1061	0.0692
*K *= 2						

*m *= 2						
*N *= 2	16	10	3	3	0.0996	0.0585
*K *= 3						

*m *= 2						
*N *= 4	16	14	9	9	0.1051	0.0996
*K *= 2						

*m *= 2						
*N *= 4	16	16	6	6	0.1040	0.0997
*K *= 3						

*m *= 2						
*N *= 8	16	16	14	14	0.1063	0.1050
*K *= 2						

*m *= 2						
*N *= 8	16	16	12	12	0.1049	0.1053
*K *= 3						

**Table 5:** A 6-Node Example for PBN With Random Perturbation

	Size		Cost		CPU Time (sec.)	
	
	Original	Reduced	Original	Reduced	Original	Reduced
*m *= 1						
*N *= 2	32	12	31	31	0.0402	0.0162
*K *= 2						

*m*= 1						
*N *= 2	32	23	10	10	0.0375	0.0257
*K *= 3						

*m *= 1						
*N *= 4	32	28	24	24	0.0396	0.0329
*K *= 2						

*m *= 1						
*N *= 4	32	30	26	26	0.0386	0.0363
*K *= 3						

*m *= 1						
*N *= 8	32	32	30	30	0.0402	0.0399
*K *= 2						

*m *= 1						
*N *= 8	32	32	30	30	0.0419	0.0418
*K *= 3						

*m *= 2						
*N *= 2	16	12	13	13	0.0315	0.0213
*K *= 2						

*m *= 2						
*N *= 2	16	15	9	9	0.0277	0.0262
*K *= 3						

*m *= 2						
*N *= 4	16	14	9	9	0.0300	0.0250
*K *= 2						

*m *= 2						
*N *= 4	16	16	7	7	0.0293	0.0292
*K *= 3						

*m *= 2						
*N *= 8	16	16	15	15	0.0316	0.0314
*K *= 2						

*m *= 2						
*N *= 8	16	16	13	13	0.0316	0.0307
*K *= 3						

### A 12-gene example

We then consider a 12-node example. We consider the cases of *m *= 1,2, *N *= 2,4,8 and *K *= 2,3. Again the Boolean function set of PBN are randomly generated. We let *M *= 40,*C*(*z_M_*) = *z_M_*. When *m *= 1, there are 11 internal nodes and 1 control node. The original network size is 2^11^. When *m *= 2, there are 10 internal nodes and 2 control nodes. The original network size is 2^10^. Table 6 gives the numerical results for PBNs without perturbation. Table 7 gives the numerical results for PBNs with random perturbation. We see that our proposed reduction method is both efficient and effective.

**Table 6:** A 12-Node Example for PBN Without Perturbation

	Size		Cost		CPU Time (sec.)	
	
	Original	Reduced	Original	Reduced	Original	Reduced
*m *= 1						
*N *= 2	2048	426	757	757	63.84	4.07
*K *= 2						

*m *= 1						
*N *= 2	2048	700	1258	1258	256.68	36.89
*K *= 3						

*m *= 1						
*N *= 4	2048	502	1830	1830	272.27	23.27
*K *= 2						

*m *= 1						
*N *= 4	2048	1462	1591	1591	264.39	143.48
*K *= 3						

*m *= 1						
*N *= 8	2048	1103	2036	2036	279.58	88.80
*K *= 2						

*m *= 1						
*N *= 8	2048	1801	1987	1987	272.35	243.79
*K *= 3						

*m *= 2						
*N *= 2	1024	350	607	607	153.85	24.35
*K *= 2						

*m *= 2						
*N *= 2	1024	444	179	179	148.80	36.10
*K *= 3						

*m *= 2						
*N *= 4	1024	415	342	342	140.62	29.49
*K *= 2						

*m *= 2						
*N *= 4	1024	801	328	328	150.21	107.27
*K *= 3						

*m *= 2						
*N *= 8	1024	759	736	736	146.98	87.76
*K *= 2						

*m *= 2						
*N *= 8	1024	937	756	756	172.93	146.29
*K *= 3						

**Table 7:** A 12-Node Example for PBN With Random Perturbation

	Size		Cost		CPU Time (sec.)	
	
	Original	Reduced	Original	Reduced	Original	Reduced
*m *= 1						
*N *= 2	2048	363	1906	1906	18.19	0.89
*K *= 2						

*m *= 1						
*N *= 2	2048	867	322	322	17.99	3.75
*K *= 3						

*m *= 1						
*N *= 4	2048	581	2005	2005	18.27	1.92
*K *= 2						

*m *= 1						
*N *= 4	2048	1340	1931	1931	18.27	8.35
*K *= 3						

*m *= 1						
*N *= 8	2048	830	1990	1990	18.42	3.59
*K *= 2						

*m *= 1						
*N *= 8	2048	1882	1847	1847	18.38	15.86
*K *= 3						

*m *= 2						
*N *= 2	1024	204	286	286	9.67	0.67
*K *= 2						

*m *= 2						
*N *= 2	1024	508	153	153	9.60	2.81
*K *= 3						

*m *= 2						
*N *= 4	1024	384	242	242	9.63	1.78
*K *= 2						

*m *= 2						
*N *= 4	1024	785	486	486	9.69	6.09
*K *= 3						

*m *= 2						
*N *= 8	1024	747	839	839	9.91	5.68
*K *= 2						

*m *= 2						
*N *= 8	1024	992	611	611	9.87	9.26
*K *= 3						

## Conclusions

From the experiment results, one can see that applying dynamic programming-based algorithm on the reduced network can reduce the computational complexity. The performance of the algorithm on the reduced network depends on the parameters of *n*, *m*, *N *and *K*. For *n *= 6, from Table 3 and Table 4, one can see that in general, there are some improvements in computational time when reduction method is applied. However, for *n *= 12, Table 6 and Table 7 indicate that when the number of nodes is large and *K *= 2, the algorithm on the reduced network performs much better than the one on the original network. Therefore, our proposed method is effective for larger size networks. Future research issues will pay attention to statistical analysis of the distribution of zero rows in transition matrix in terms of *n*. Moreover, we will keep exploring ways of reducing computational complexity of intervention strategies.

## Competing interests

The authors declare that they have no competing interests.

## Authors' contributions

XC came up with the idea. XC and WKC designed the research. XC, HJ and YQ performed the research and analyzed the results. XC, HJ and WKC wrote the paper. All authors read and approved the final manuscript.
